# Adaptation of Inuka coaching problem-solving therapy to support mental health and HIV medication adherence among status-neutral men who have sex with men in South Africa

**DOI:** 10.1093/inthealth/ihaf086

**Published:** 2025-08-11

**Authors:** Lindiwe Tsope, Elise M van der Elst, Jacqueline Pienaar, Charlene Denousse, Mapaseka Mabena, Pontsho Komane, Boitumelo Ramashala, Ankiza Gakunu, Danielle Giovenco, Ruwenne Moodley, Don Operario, Eduard J Sanders

**Affiliations:** Implementation Science Division, The Aurum Institute, Johannesburg 2194, South Africa; Department of Interdisciplinary Social Science, Utrecht University, Utrecht, The Netherlands; Implementation Science Division, The Aurum Institute, Johannesburg 2194, South Africa; Inuka Foundation, Nairobi, Kenya; Implementation Science Division, The Aurum Institute, Johannesburg 2194, South Africa; Implementation Science Division, The Aurum Institute, Johannesburg 2194, South Africa; Implementation Science Division, The Aurum Institute, Johannesburg 2194, South Africa; Inuka Foundation, Nairobi, Kenya; Behavior, Social and Health Education Sciences, Emory University, Georgia 30322, USA; Behavior, Social and Health Education Sciences, Emory University, Georgia 30322, USA; Behavior, Social and Health Education Sciences, Emory University, Georgia 30322, USA; Implementation Science Division, The Aurum Institute, Johannesburg 2194, South Africa; Dunn School of Pathology, University of Oxford, Oxford OX1 3RE, UK

**Keywords:** depression, Inuka coaching, medication adherence, mental health, MSM, South Africa

## Abstract

**Background:**

Depression is prevalent among men who have sex with men (MSM) in South Africa and can impact their adherence to PrEP and ART. We developed the WeCare coaching intervention, adapted from the evidence-based Friendship Bench/Inuka problem-solving therapy program, to address their mental health challenges.

**Methods:**

Twenty MSM with symptoms of mild to moderate depression (Patient Health Questionnaire-9 scores 5–14) and using PrEP or ART were recruited from two clinics in Johannesburg and Pretoria. Participants received adapted Inuka coaching (four individual and up to four group sessions). Mental health outcomes were assessed preintervention and postintervention using the SRQ-20. Postintervention in-depth interviews and focus group discussions were conducted with participants and lay health workers (LHWs). Qualitative data evaluated utility. Data were analysed using thematic analysis.

**Results:**

Median SRQ-score improved from preintervention to post-intervention assessment (p<0.001). Participants found coaching acceptable, qualitatively highlighting the value of a safe space to discuss emotional well-being. Key themes included: (1) need for cultural and contextual adaptation of coaching content; (2) stigma-related stressors shaping mental health and perceived engagement with ART/PrEP; and (3) LHWs expressed enthusiasm for delivering support but requested strengthened ART/PrEP literacy training.

**Conclusion:**

Findings supported acceptability and feasibility and informed further development of the WeCare intervention.

## Introduction

South Africa (SA) has the largest HIV epidemic globally, with an estimated 8 million people living with HIV.^1^ The epidemic is generalised, affecting the population at large, but continues to present distinct vulnerabilities among specific groups. Within this broader context, men who have sex with men (MSM) represent a priority population for HIV prevention and treatment efforts. While surveillance data remain limited, available evidence suggests that MSM in sub-Saharan Africa (SSA), including SA, experience elevated risks of HIV acquisition compared with the general male population.^2,3^ These disparities are shaped by overlapping structural and social factors, including stigma, discrimination and reduced access to tailored health services.^4^

In a systematic review of data from 22 SSA countries, MSM were found to have some of the highest estimated incidence rates of all key populations, although the reliability of comparative data remains limited by undersampling and under-reporting.^5^ In SA, MSM face a range of challenges that intersect with HIV risk and care engagement, including high levels of depression, internalised homophobia and anticipated stigma in healthcare settings.^5,6^ Despite this elevated need for services, few empirically supported interventions address the combined mental health and HIV prevention and treatment needs of MSM in this region.^7^

Mental health challenges are common among MSM in SA and may impact antiretroviral treatment (ART) and pre-exposure prophylaxis (PrEP) medication adherence.^6^ A recent survey of 300 MSM in SA reported that more than one-half met the criteria for clinical depression, and that higher depression scores were significantly associated with factors including internalised homonegativity, enacted and anticipated MSM stigma.^7^ Common mental health disorders, including depression and anxiety, among MSM in SA have been attributed to minority stress responses related to anti-MSM stigma.^8^ According to the minority stress model, internalisation of MSM-related stigma produces a range of adverse biological, behavioural and clinical health reactions, including poor mental health.^9^

There is emerging literature in SSA supporting the beneficial effects of mental health interventions for common psychosocial problems and HIV medication adherence.^10,11^ The Friendship Bench (FB) is an evidence-based community mental health intervention developed and validated with general populations experiencing mental health problems in multiple low-resource settings in SSA.^12–14^ A key feature of the FB is the use of lay health workers (LHWs) who are trained to deliver cognitive problem-solving therapy for individuals screening positive for depression and anxiety.^15^ FB participants are also invited to join peer support groups. In a trial of the FB programme in Zimbabwe, participants recruited from the general community received up to six sessions of individual problem-solving therapy delivered by trained and supervised LHWs, and were offered an optional four to six sessions of a peer-support programme.^14^ Among 573 randomised participants, including 41.7% participants living with HIV, the intervention group had a lower risk of symptoms of depression (13.7% vs 49.9%; adjusted risk ratio, 0.28; 95% CI 0.22 to 0.34) after 6 mo of follow-up.^16,17^ Subsequent studies found that a mobile/internet-delivered version of the FB (referred to as the ‘Inuka coaching’ approach) was also associated with fewer mental health problems and had comparable levels of engagement and retention as in-person delivery.^18,19^

We developed a programme entitled *WeCare* based on the FB and Inuka intervention protocols to address the dual and interconnected priorities of mental health and medication adherence problems among MSM in SA.^20^ The programme uses an HIV status-neutral approach to include both MSM living with HIV and who are using PrEP as well as MSM not living with HIV who are using PrEP. In this paper, we describe our experience piloting the *WeCare* programme and report postintervention experiences of participants and certified MSM LHWs. This description and preliminary findings from this pilot study offer a framework for ongoing Inuka coaching adaptation for MSM in SA.

## Methods

### Study setting

The intervention was provided at the Aurum Institute, a non-profit organisation that offers HIV support services to >20 000 key population members at five ‘Pop Inn’ clinics in SA, including at clinics in Tshwane (Pretoria) and Ekurhuleni (Johannesburg) that were used for the current study. Pop Inn clients receive free PrEP or ART medication, adherence counselling, 3-monthly testing for HIV (when on PrEP), screening for sexually transmitted infections and TB, as well as psychosocial support services. Clients regularly participate in monthly peer-led ‘mpowerment’ group meetings of 8–10 MSM, where group members talk about their general challenges and health-related experiences. The clinics also provide free Wi-Fi, access to computers and a comfortable space to relax and connect with peers.

### Study participants and intervention description (*WeCare* programme)

From July to October 2023, active LHWs were selected from MSM peer-support groups (‘mpowerment sessions’) at Pop Inn clinics in Johannesburg and Pretoria. Individuals who were eligible to serve in the role of LHWs were self-identified MSM who demonstrated consistent engagement with clinic services, strong peer communications skills and a personal interest in promoting mental health and HIV care in their communities. The final selection of LHWs was conducted collaboratively by clinic staff and programme coordinators, with an emphasis on candidates’ emotional maturity, reliability and willingness to uphold confidentiality and non-judgemental support.

Individuals selected to serve as LHWs completed a structured, multiphase training programme adapted from the Inuka digital coaching model and the FB problem-solving therapy approach. The training comprised 4 d of supervised online instruction, 5 d of self-directed learning and module review, followed by a 4-wk period of practical training through chat-based sessions. During this practicum phase, trainees completed nine supervised coaching sessions (three initial sessions, three follow-up sessions and three sessions addressing high-risk cases including suicidal ideation) and participated in seven sessions as coaches to develop empathy and insight into the user experience.

The curriculum covered Inuka's six-step coaching framework, foundational mental health principles and core aspects of research and professional ethics. Practical components included role-play exercises and counselling simulations, with targeted feedback on active listening and problem-solving techniques. LHWs were required to demonstrate competence through mock sessions and a practice examination. Upon successful completion of all training components, participants were awarded a certification of completions for the Inuka coaching programme. Ongoing supervision and performance support were provided throughout the training period to reinforce skill development and ensure intervention fidelity.

Over the course of 3 mo of pilot programme implementation, the five LHWs delivered four individual and up to four group coaching sessions to a total of 20 MSM enrolled programme participants. Session delivery and coaching performance was supervised and supported by a professional Inuka trainer. LHWs received feedback based on review of their coaching transcripts and recordings.

The 20 participants were selected from a recent cross-sectional survey conducted at the same study sites (Pienaar et al., 2025)^21^, based on Patient Health Questionnaire-9 (PHQ-9) scores of 5-14 indicating mild to moderate depression.

#### *WeCare* intervention description

The *WeCare* intervention is an adaptation of the Inuka/FB problem-solving therapy model, tailored specifically for use with MSM in SA. Building on the original Inuka model, *WeCare* was adapted to incorporate coaching support for the context-specific challenges faced by status-neutral MSM.

The *WeCare* intervention entails cognitive behaviour therapy coaching in practical steps. Guided by the MSM participant's stated needs, the LHW comes up with actional steps toward improvement of a self-identified challenge. The core components of the intervention consist of (i) identifying the problem; (ii) exploring the individual's experience and manifestations of the problem; (iii) brainstorming feasible solutions and options available; (iv) setting a specific, measurable, achievable and realistic action plan; (v) implementing the action plan; and (vi) providing reassurance and follow-up.

The intervention comprised four individual coaching sessions per participant, and were offered online or in-person depending upon the participant's choice. Participants were also invited to take part in Inuka's group coaching sessions. In preparation for the group coaching sessions, LHWs identified commonly shared challenges based on their individual discussions with participants (Supplementary Table 1) and prioritised the three topics most frequently mentioned for the group sessions (Supplementary Table 2). Because medication adherence challenges were infrequently mentioned by coaches, we added this as a fourth topic due to the focus of the project.

### Data collection

Data about sociodemographic and mental health characteristics were obtained at enrolment. Participants completed the self-reporting questionnaire (SRQ-20) prior to the start of their first individual session and immediately after completion of their final individual session. The SRQ-20 is a validated 20-item psychological screening tool that is used to assess mental health and well-being, with higher scores indicating greater mental distress.^22^

In-depth open-ended qualitative interviews (IDIs) were conducted upon completion of the Inuka coaching intervention with MSM participants and the five LHWs. Four focus group discussion (FGD) feedback sessions were conducted with study participants and one was conducted with LHWs. IDIs and FGDs were conducted in English, in person or online based upon participants’ choices, digitally recorded and transcribed verbatim by an independent transcriber. IDIs lasted about 45–60 min and FGDs about 60–90 min. A reflective summary of each IDI and FGD was produced upon completion of each session.

### Data analysis

Preintervention and postintervention SRQ-20 scores were compared using the Wilcoxon signed-rank test, a non-parametric method suitable for small paired samples and ordinal outcome data.

Qualitative data were analysed thematically using the Braun and Clarke^23^ reflexive approach. Coding was conducted inductively by three independent researchers to identify patterns, generate preliminary codes and construct themes. To enhance analytical rigour, the team engaged in ongoing memoing to document reflexive insights and analytic decisions throughout the coding process. Peer debriefings and consensus discussions were held to resolve coding discrepancies and strengthen interpretation. Findings were triangulated across individual interviews and focus groups, and member checking was conducted during a participatory validation workshop with MSM participants to confirm and refine key themes.

### Ethical considerations

The University of the Witwatersrand Human Research Ethics Committee reviewed and granted ethics approval for this study (AUR 8–403). All participants provided written informed consent for participation, audio recording of IDIs and FGDs and use of their quotations. All participant records and information were anonymised and deidentified prior to analysis.

## Results

### Quantitative findings

Of 20 participants, three did not continue coaching following their intake visit due to various personal and professional commitments, including conflicting work and study schedules, inability to attend sessions due to work demands and a personal decision to delay participation until a later time when they deemed coaching necessary. A description of the characteristics of the 17 participants is shown in Table 1. There were no statistically significant differences in characteristics of MSM who used PrEP or ART. The median age was 31.9 y and all participants were black Africans. The majority (64%) had attained tertiary education, 73% had some form of employment, 24% had moderate symptoms of depression and 76% reported having medication adherence challenges, defined as missing a dose of their medication on ≥1 d in the last month.^21^ Six participants were on anti-retroviral therapy (ARVs) with a median duration of 5.4 y and 11 participants were on PrEP with a median duration of 1.7 y (data not shown).

**Table 1.** Characteristics of men who have sex with men participating in the Inuka coaching evaluation, Johannesburg and Pretoria, 2023

	Overall (n=17)		PrEP (n=11)		ART (n = 6)	
Characteristic	n (%) or median (IQR)		n (%) or median (IQR)		n (%) or median (IQR)	
Age (y)	31.9 (28.0–36.9)		31.9 (24.4–36.9)		31.9 (28.0–37.7)	
Education						
Primary	2	(12)	2	(18)	0	(0)
Secondary	4	(24)	4	(36)	0	(0)
Higher/tertiary/other	11	(64)	5	(45)	6	(100)
Employment						
Unemployed	6	(27)	4	(36)	2	(33)
Employed (full time)	8	(53)	5	(45)	3	(50)
Employed (part time/casual)	1	(7)	0	(0)	1	(17)
Self-employed	2	(13)	2	(18)	0	(0)
Race						
Black African	17	(100)	11	(100)	6	(100)
Depressive symptoms (PHQ-9)^a^						
Minimal to mild (0–9)	13	(76)	9	(82)	4	(67)
Moderate (10–14)	4	(24)	4	(18)	2	(33)
Disordered alcohol use (AUDIT), past year						
Low (0–7)	11	(65)	8	(73)	3	(50)
Hazardous (8–40)	6	(35)	3	(27)	3	(50)
Medication adherence						
Miss taking PrEP/ART on ≥1 d	13	(76)	9	(82)	4	(67)
Site						
Pretoria	9	(53)	5	(55)	4	(67)
Johannesburg	8	(47)	6	(45)	2	(33)

ART: antiretroviral therapy; AUDIT: Alcohol Use Disorder Identification; PHQ-9: Patient Health Questionnaire-9; PrEP: pre-exposure prophylaxis.

^a^PHQ-9=only participants with mild to moderate symptoms of depressions were selected.

#### Preassessment and postassessment scores

Participants’ median SRQ-score decreased from 8 to 2 (p<0.001) with no indication of clustering at zero change, suggesting that the intervention had improved well-being from preintervention to postintervention (changes in individual scores are listed in Supplementary Table 3).

### Qualitative findings

A total of 20 IDIs and three FGDs took place. We conducted IDIs with 15 of the 17 participants who completed the programme; of these, five participated in a feedback FGD session. In addition, all five LHWs participated in an IDI and in a feedback session.

Informed by the Clarke and Braun thematic approach for qualitative data,^23^ we identified three main thematic areas to guide further development and integration of the adaptation of the *WeCare* intervention for this population: (i) Inuka coaching was acceptable to MSM participants, but requires adaption to their context and needs; (ii) ART/PrEP adherence and mental health challenges were impacted by anticipated stigma, including internalised homophobia and concerns about sexuality and HIV disclosure; and (iii) LHWs indicated enthusiasm for acquired counselling skills and wanted to strengthen their knowledge of ART and PrEP adherence.

### Inuka coaching was acceptable to MSM participants but requires adaptation to their context and needs

Participants emphasised the utility of this coaching approach as a platform to discuss mental health issues, which they would ordinarily not discuss:

*Yeah, well…I've never had conversations with anyone regarding my personal life. So I grew up knowing that as a man you have to find a way. So [Inuka coaching] made me feel that maybe it doesn't hurt to talk about these kind of things. And it actually worked* (PID_09, IDI).

LHWs emphasised the need for continued mentorship and continual recognition of mental health issues as these were seen as silent issues. However, the next quote suggests that, for the therapy to be effective, it needs to resonate with the participants’ cultural background:

*…in black communities, therapy counselling is always disputed. They will tell you ‘it is a waste of time, a waste of money’, but it is very helpful, you get to be listened to and supported at a professional level* (LHW_1, FGD).

Inuka's focus on each participant's individual characteristics and preferences demonstrated to recipients that taking care of their health directly could lead to overall quality of well-being, as noted in this quote:

*I can recommend [Inuka] coaching to anyone who is going through a lot…the coaching showed me how important is it that I take care of myself. My health is priority in life* (PID_15, IDI).

Participants highlighted that ‘being coached by another MSM’ promoted a safe space and sense of camaraderie. They felt that the LHWs respected them and understood the nuances of being an MSM in this social/cultural context:

*I felt like I am with someone who understands, who has been through this situation that I'm going through…So, I felt like I was speaking with someone who was listening to me* (PID_12, IDI).

Both participants and LHWs felt that the Inuka programme should be incorporated as the standard of care for MSM seeking services at private and public facilities:

*I think these sessions should continue—not with us who have already been part of this—but for new people to learn what I have learned* (PID_08, IDI).

Participants voiced a belief in the intervention's value for the MSM community as a whole, which they noted can improve the well-being of future generations of LGBTQI+ people:

*…We are still trying to educate our community about this LGBTQI+ people, and who we are. We have these young kids who are part of the LGBTQI+ community, but they are scared to come out. Maybe this programme can allow them to be themselves* (PID_03, IDI).

### ART/PrEP adherence and mental health challenges were impacted by anticipated stigma, including internalised homophobia and concerns about sexuality and HIV disclosure

Participants described how experiences of stigma, internalised homophobia and fears related to sexual identity and HIV disclosure negatively influenced their mental well-being. Participants perceived that these stressors contributed to their difficulties with maintaining ART or PrEP adherence, even although adherence itself was not quantitatively assessed in the preassessment and postassessment. This shared perception aligns with our prior work conducted among MSM in Johannesburg and Pretoria, which found that more than one-half (53.5%) of PrEP- and ART-taking MSM had adherence challenges, and that moderate to severe depressive symptoms (PHQ-9≥10) were significantly associated with missed doses (adjusted prevalence ratio=1.50, 95% CI 1.19 to 1.89). These prior findings align with the qualitative accounts shared by current *WeCare* participants, who identified emotional distress and stigma as barriers to consistent medication use.

Participants’ concerns and fears about their HIV-positive status contributed to a negative self-image and impacted their adherence to treatment and disengagement coping. The programme’s deliberate effort to recognise participants’ experiences of HIV-related stigma helped them to develop resilience related to HIV-status stressors. Skills to develop self-compassion and promote self-care and adherence were noted in this quote:

*I'd really love this [Inuka program]…because there is reassurance from the Inuka coach to take care of myself and emphasis on taking my medication again* (P_2, FGD).

In terms of disclosure of sexual identity, participants shared that they anticipated prejudice and discrimination in their families, communities and society as a whole. For them to constantly ‘come out’—as it is not a one-time event—is often extremely stressful, especially in unfamiliar situations:

*I was in the closet, I was afraid the family would reject me, to become one of those who was going to be discriminated against, to be hurt or do things to me…* (PID_07, IDI).

 

*…family expectations, what can I say, to be free with who you ARE [gay] in this world of today. The family is worried what the people in the community will say or think* (PID_15, IDI).

Participants called for personal and societal paradigm changes. They felt that individuals who are resistant to altering established practices may hinder the intervention's implementation efforts:

*…I think, as youth, we were deprived the chance of talking, how we feel…When it comes to parents, some rural, some old…they don't understand, nor do they know about these ‘LGBTI-things’. They are too stereotyped and stubborn* (PID_10, IDI).

The above quotes succinctly capture the disclosure challenges associated with stigma experiences and mental health issues within the MSM community. Addressing these challenges requires a multifaceted approach within a supportive environment, where people feel safe and respected.

### Coaches indicated enthusiasm for acquired counselling skills and wanted to strengthen their knowledge of ART/PrEP adherence

During post-training focus groups, LHWs described how the Inuka training had influenced their ongoing ability to coach their MSM peers. They mentioned transformations in their capacity, integrating new knowledge and guiding skills:

*Inuka provided us with messages and guidance to help our clients make problems more manageable. We feel more confident now we are trained and better equipped* (LHW 4, feedback session 3).

LHWs recognised that their newly acquired skills of focusing on participants’ comfort and addressing expressions of distress are an essential attribute to delivering the *WeCare* programme. Through Inuka's training, LHWs felt stronger confidence in their ability to execute the coaching:

*…They [Inuka] gave us a lot of tools. Even if the client is not okay, there are techniques to calm the client down* (LHW_5, IDI).

LHWs repeatedly highlighted a key feature of Inuka's training: ‘the importance of creating a safe space to enable and guide their clients through identification of mental issues’. LHWs fostered this safe space through befriending and bonding with participants in a professional manner:

*I [as a coach] mainly focus on building a great relationship, a ‘translated relationship’ with my client, even when I am not [physically] in the client's space, I am there for them* (LHW_2, IDI).

 

*We [as LHWs] ensure the client that there's confidentiality. We create a rapport. We also made clients feel free to share more…to make the clients feel more comfortable around us to say whatever they want to say they want to share with us without any fears* (LHW_3, FGD).

During the feedback sessions, LHWs remarked that improved knowledge and enhanced skills about the technical issues related to supporting ART and PrEP adherence were especially needed at the precoaching level to more easily recognise and emphasise clients’ adherence challenges:

*We [as coaches] need more information about ARVs and PrEP ourselves…because when a client is struggling with adherence, we need to be able to understand why and how we can guide the client better* (LHW 2, feedback session 1).

LHWs recommended that an ‘adherence module’ should be integrated in the Inuka training to support MSM taking ART or PrEP. They also noted that future training sessions should develop LHWs’ knowledge about multiple antiretroviral drugs as well as provide detailed information on how PrEP works, as these are key topics that future LHWs must provide to participants. According to them, medication literacy could empower them to potentially make better decisions:

*Can we be educated with different types of ARVs? How PrEP works, when to take PrEP, when to take ARVs…What are the good times? What are the good and the not good. So, education on the medication I think it's very vital* (PID_08, IDI).

LHWs mentioned that having greater knowledge about HIV medications will help them to respond to the unique and varied needs of MSM participants, and that this improved professional understanding would result in stronger acceptability and effectiveness of ART and PrEP treatment in their community:

*I sometimes have a problem taking my medication. It's just that I was lacking information when it comes to taking medication. Yeah, that's what I learned and I discovered when I was attending* [the Inuka sessions] (PID_15, IDI).

Collecting information on LHWs’ and participants’ experiences with the Inuka training intervention, coupled with MSMs’ insights into medication non-adherence, allowed for enhanced adaptation of Inuka's coaching problem-solving therapy, which is essential to support mental health and HIV medication adherence among status-neutral MSM in SA.

## Discussion

We found preliminary evidence supporting the *WeCare* programme as an acceptable approach to providing mental health support for MSM living with or at risk for HIV in SA, and we also found areas needing further adaptation to optimise relevance and impact on programme recipients. In addition to the potentially positive impact on participants’ mental health, we observed promising feedback from LHWs about having improved competencies towards delivering peer-based coaching to MSM programme recipients, along with domains that need strengthening for future training. In particular, LHWs noted a need for skills to address and de-escalate participants’ psychological distress that might arise during counselling, as well as deeper content knowledge about HIV medications to support participants’ medication adherence. Likewise, community-based MSM who are the intended programme recipients commented favourably regarding the personalised problem-centred counselling approach that affirmed their sexual identity, but also suggested that receiving additional coaching and education about the importance of medications could benefit the programme.

The pre-post SRQ-20 assessment detected improvements among participants in mental health symptoms by the time of the final individual counselling session. Despite the study's small sample size, our pilot study suggests a promising potential for the *WeCare* programme to address the high levels of stress, anxiety and depression that have been previously observed in this population, and which can impact medication adherence. More rigorous evaluation with longer follow-up and inclusion of a comparison group (e.g. standard of care) can provide a more rigorous evaluation of this approach.

Qualitative analyses provided essential additional information to improve efforts to enhance LHWs’ capability to provide peer mental-health coaching to their peers. Key actions for improved training were identified, including the need to incorporate tailored coaching therapy to MSM and improving LHWs’ skills in addressing participants’ displays of psychological distress, as well as adjusting language, metaphors and techniques to improve relevance to the local MSM culture. In addition, LHWs’ feedback indicated a need to design a stand-alone module to improve HIV medication literacy when counselling MSM (module provided as supplementary material).

Exploration of participants’ HIV medication adherence challenges yielded insight into a complex interplay between their mental health, HIV-related and sexual-orientation stigma, insufficient medication knowledge and adherence behaviour. As described by Pienaar and colleagues,^21^ depression among MSM in SA is common and a major risk factor for poor medication adherence. A systematic review and meta-analysis showed an estimated prevalence of depression among people living with HIV who are on ART in eastern and southern Africa of 38% (95% CI 27 to 49%).^24^ Findings from this study corroborated that the medication adherence among MSM may be impacted by the presence of multiple co-occurring conditions, including depression and anxiety, internalised homophobia, trauma and post-traumatic stress disorders, substance use, sexual compulsivity and risk behaviours.^7^

Integrating mental health screening at points of HIV care can enable linkages to individual and group counselling, support groups and peer support activities for MSM where they can discuss emotional and psychological issues that might also impact treatment adherence,^25^ including viral load suppression.^26^ For PrEP-taking MSM, greater connection and group support have been shown to improve PrEP adherence.^27,28^

**Figure 1.**
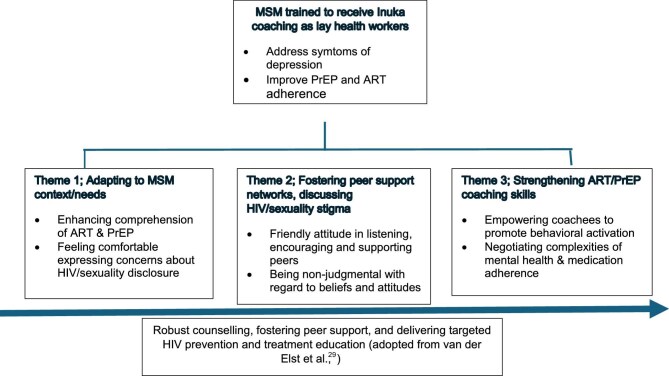
Conceptual framework highlighting three key themes of the *WeCare* adaptation to facilitate coaching of status-neutral men who have sex with men (MSM) with medication challenges and depression in South Africa. ART: antiretroviral treatment; PrEP: pre-exposure prophylaxis.

We incorporated these findings into an enhanced conceptional framework to guide further development and evaluation of the *WeCare* training programme for improving LHWs’ capacity to address mental health and medication adherence among MSM (Figure 1).^29^ The conceptual *WeCare* training framework highlights the importance of psychological, social and treatment challenges faced by status-neutral MSM.

Drawing on participant feedback, LHWs’ insights and coaching observations, several additional adaptations were identified to strengthen the relevance and effectiveness of the *WeCare* intervention for MSM in SA: (i) to include cultural and linguistic adaptation, such as examples to reflect local languages, idioms and lived experiences of MSM to ensure that coaching resonates with participants’ cultural and social realities; (ii) to enhance the focus on stigma navigation, expanding coaching scenarios to include guided conservations on navigating internalised homophobia; (iii) to tailor the medication literacy module, integrating simplified instructions, for example, affirming education on PrEP, ART and dosing schedules; (iv) to actively discuss each client's medication adherence, incorporating a structured adherence tool (e.g. the visual analogue scale^30^); (v) to introduce flexible coaching delivery formats, offering hybrid options (online and face-to-face) to accommodate participants’ preferences; (vi) to strengthen coach supervision and peer learning, including regular debriefs and emotional support to help LHWs navigate complex client situations; (vii) to expand group coaching modules reinforcing peer support, reducing isolations and facilitating collective coping strategies for stigma and adherence challenges; and, finally (viii) to equip coaches with clear referral pathways for mental health crises, alongside ethical training to maintain boundaries and confidentiality. Recognising the boundaries of LHWs’ roles and ensuring timely referral to professional counselling services when a client’s needs exceed their training or scope is particularly essential, especially in cases of severe psychological distress, trauma, risk of harm or suicidal ideation.

Based on this enhanced training of LHWs, our adapted *WeCare* intervention will be more likely to foster programme recipients’ comprehension of their personal action plans, including actions to improve medication adherence and normalise open discussions about HIV and sexuality-related stigma in individual and group sessions (see the supplementary material: Inuka *WeCare* adherence facilitation for MSM). Rigorous evaluation is needed to determine programme effectiveness on these intended outcomes, and further implementation analyses must determine the necessary factors to optimise adoption and sustainability of this intervention approach in community or clinical settings where MSM seek services.

Our study has limitations. First, this was a small study with English-speaking participants, of whom the majority had completed secondary or higher education, limiting the generalisability of our findings. Second, the study was conducted at an established HIV service organisation that provided programmes to MSM and offered free daily PrEP and ART; although this is an aspirational context for many low- and middle-income settings, the findings reported here may not generalise to all South African MSM. Finally, we were unable to interview participants who dropped out from the study and their reasons for not participating may be important to understand.

### Conclusion

The *WeCare* intervention, adapted from the Inuka coaching intervention and the FB problem-solving therapy model, offers a new status-neutral approach to provide mental health support and address perceived barriers to ART and PrEP adherence, including stigma, internalised homophobia and limited medication literacy among MSM in a low-resource setting.

## Supplementary Material

ihaf086_Supplemental_File

## Data Availability

The data that support the findings of this study are available from the corresponding author, EJS, upon reasonable request and approval of principal investigators.
