# Supplementary material for: Adaptation of Inuka coaching problem-solving therapy to support mental health and HIV medication adherence among status-neutral men who have sex with men in South Africa
Source: Int Health. 2025 Aug 11;18(2):245–53. doi: 10.1093/inthealth/ihaf086 (PMC13016760; doi:10.1093/inthealth/ihaf086)
Supplement: ihaf086_Supplemental_File [file ihaf086_supplemental_file.pdf]

Supplemental table 1: Challenges mentioned at start of Individual Inuka coaching sessions by 20 status neutral participants with mild to moderate symptoms of depression, Johannesburg and Pretoria, 2023

**Ekurhuleni (Johannesburg)**		**Tshwane (Pretoria)**	
Problem	Number of times[1]	Problem	Number of times[2]
Relationship problems	6	Relationship problems	7
Stigma from family & community	4	Stigma from family & community	6
Financial problems	2	Financial problem	5
Job / work / school problems	2	Job/work/school problem	4
Emotional, physical, sexual abuse	1	Emotional, physical, sexual abuse	3
HIV status disclosure	1	Drug and alcohol use	2
1). Frequency of problem named in 25 Inuka coaching sessions		2). Frequency of problem named in 34 Inuka coaching sessions.	

Supplemental table 2: Inuka group session discussion themes and prompts

**Group 1**	**Relationship challenges**
Prompts	• What relationships are important/ toxic?<br>• What are the relationship challenges?<br>• How do you recognize a relationship challenge?<br>• What are the triggers that may cause a relationship challenge?<br>• How do relationship challenges impact HIV status disclosure?<br>• How do relationship challenges impact PrEP or ART adherence?
**Group 2**	**Financial difficulties**
Prompts	• What are your views with regards to today's topic?<br>• What do you think are the common causes for financial difficulties?<br>• What are the possible solutions that can assist in addressing financial difficulties?<br>• How can financial difficulties prevent us from taking our treatment (PrEP or ART)?
**Group 3**	**Stigmatization**
Prompts	• What are your views with regards to today's topic?<br>• How have you experienced stigma in your life?<br>• How can stigma affect mental health well-being?<br>• How may stigma affect PrEP and ART medication adherence?<br>• What events or experiences may trigger the feeling of being stigmatized?<br>• What can be the possible solutions to prevent (the experience of) stigma?<br>• How may recognition of stigmatization help you to take your PrEP or ART better?
**Group 4**	**HIV status disclosure, HIV prevention, and adherence**
Prompts	• What are your views with regards to today's topic?

	• What has mental health to do with status disclosure, prevention, and adherence?<br>• Why is it important for one to disclose their HIV status?<br>• How can you disclose to your partner, friends, family, etc.?<br>• How knowledgeable is the community about HIV and what work needs doing?<br>• What are different ways that one can disclose?<br>• What do you understand by the term Undetectable= Untransmutable?<br>• How can medication adherence be supported?<br>• What can prevent one from taking their ART/PrEP?

Supplemental table 3: Inuka coaching SRQ-20 start and end of individual session scores, Ekurhuleni, and Tshwane, 2023

Participant ID	Age in years	PrEP / ART	SQR-20 start Individual session	SQR-20 end Individual session	Ranked end scores
01	38	ART	17	5	11
02	35	PrEP	15	11	10
03	41	ART	14	0	5
04	29	ART	13	5	5
05	38	PrEP	12	1	5
06	46	PrEP	11	10	5
07	31	PrEP	9	0	4
08	23	PrEP	8	0	3
09	42	PrEP	**8**	0	**2**
10	23	PrEP	8	5	1
11	25	PrEP	7	3	1
12	32	ART	7	2	1
13	33	PrEP	7	1	0
14	28	ART	7	0	0
15	34	PrEP	5	4	0
16	31	PrEP	4	5	0
17	34	ART	3	1	0

MODULE X: HIV/SEXUALITY DISCLOSURE AND ARVs & PrEP FOR MSM_06_Feb_2025

Hello there Guide! Here's a summary of supportive messages and guidance to help your client with HIV navigate the process of disclosure. These messages can help make the process of disclosure feel more manageable, supportive, and empowering, emphasizing that it is an individual choice and that support is available.

1. Understanding Disclosure Benefits:

- *"Sharing your HIV status with someone you trust can provide emotional support and strengthen your relationships."*
- *"By disclosing, you can help educate others about HIV and reduce stigma, creating a more supportive environment for yourself and others."*

2. Preparation for Disclosure:

- *"Consider starting by sharing with someone you trust and feel comfortable with; it could be a close friend, family member, or partner."*
- *"Think about why you want to disclose and what you hope to achieve. This clarity can make the conversation easier and more meaningful."*
- *"If you're worried about the reaction, practice what you'll say or even write it down to organize your thoughts."*

3. Managing Expectations:

- *"Understand that people may need time to process. Be prepared for different reactions and know that you can provide resources or support if needed."*
- *"Stay calm and clear. Focus on what you need from the person you're telling, whether it's understanding, support, or privacy."*

4. Focus on Your Well-Being:

- *"Remember, disclosure is your choice. You have the right to decide if, when, and with whom to share your HIV status."*
- *"Seek support from counseling services, peer groups, or healthcare providers if you need additional guidance on disclosure."*

5. Empowerment and Self-Care:

- *"Disclosing your HIV status can be an empowering step towards self-acceptance and confidence in managing your health."*
- *"Consider focusing on the positive aspects of your health journey, like adhering to treatment and protecting those around you."*

The following case studies can be tailored to the unique experiences of MSM living with HIV, helping them manage both mental health and HIV-related challenges effectively:

Case study 1: Non-disclosure and being HIV positive

1)Exposure Therapy

Goal: Reduce anxiety and avoidance behaviors related to HIV stigma and/or disclosure.
Situation: Sifiso is a 28-years old openly gay man. He got diagnosed with HIV some 8 years ago. As a sex worker he is the breadwinner at home. His clients are foreign African guys who don't ask for any protection. As this pays better Sifiso has remained quiet about his status. Also, Sifisio hasn't disclosed his HIV status to his long-time partner. He hides his pills in a vitamin bottle, but especially during depressive episodes, he misses ART doses. The last time his viral load was tested, it had gone up dramatically.
Sifisio is very anxious disclosing his status to his main partner of six years. He is worried that when his partner finds out, he will leave him as he kept his secret for such a long time.

- **Technique:**
    - **Gradual Exposure:** Start with imagining disclosing his status in a safe environment. Progress to practicing disclosure in role-play sessions with the therapist.
    - **Real-Life Exposure:** Gradually move towards real-life disclosure, starting with close friends or support group members.
    - **Reflection and Reinforcement:** After each step, discuss the experience and reinforce positive outcomes and coping strategies.

2)Relapse Prevention Planning

Goal: Prevent lapses in ART adherence due to mental health issues.

- **Technique:**
    - **Identify Triggers:** Identify situations, thoughts, or feelings that have led to missed doses in the past (e.g., feeling hopeless or unmotivated).
    - **Develop a Coping Plan:** Create a step-by-step plan to manage these triggers, such as setting reminders, involving a friend for accountability, or practicing self-compassion techniques.
    - **Implement Support Systems:** Establish regular check-ins with a healthcare provider or support group to monitor adherence and mental health.

Case study 2: HIV status disclosure, and being on ART

Problem-Solving Therapy

Goal: Enhance problem-solving skills to reduce feelings of overwhelm and helplessness.
Situation: Bandile is a gay man and HIV positive for ten years. He feels supported by his mother and four sisters who are close to him and who know that he is gay. He takes his tablets every day and his viral load tests of the last 4 years show that his virus is undetectable. He has a boyfriend of 5 years. They have unprotected intercourse, but Bandile

has not disclosed his status as he fears that his boyfriend will leave him when he finds out. More and more he feels overwhelmed by managing his health, mental health, and relationship.

- **Technique:**
    - **Problem Identification:** Help him clearly define the problems he is facing.
    - **Generate Solutions:** Brainstorm possible solutions, even unconventional ones, without judgment.
    - **Evaluate and Choose Solutions:** Assess the pros and cons of each solution and choose the most feasible.
    - **Implement and Review:** Encourage him to implement the chosen solution and review its effectiveness, adjusting the plan as needed.

Dear Guide, here are some brief and supportive messages that may help with disclosing sexuality as an MSM. Each of these messages can help communicate openness, trust, and a desire for continued understanding and support.

1. Be direct and clear:

- *"I want to share something important with you about myself. I'm attracted to men, and this is a part of who I am."*
- *"I trust you and want to be open with you. I'm a man who is attracted to men, and I hope you can support me."*

2. Affirming Openness:

- *"I value our relationship and feel safe enough to be honest with you. I'm a man who has relationships with other men, and I hope this won't change how you feel about me."*
- *"This is a part of myself that I want to share with you, as someone I care about. I'm attracted to men, and being open about this is important to me."*

3. Expressing Vulnerability:

- *"I've wanted to share this for a while because it's a big part of who I am. I'm attracted to men, and your support would mean a lot to me."*
- *"It's taken me time to be comfortable with this part of myself, and I'm hoping you'll understand and be there for me."*

4. Setting Positive Expectations:

- *"I'm telling you this because I trust you and know that you care about me. I'm a man who has romantic relationships with other men, and I hope you can support me as I am."*
- *"Being open about this is important to me, and I hope we can continue to have a positive relationship with mutual respect."*

5. Inviting Understanding:

- *"I know this might be new information for you, but I'm here to answer any questions or talk more if you need."*
- *"I understand if you need time to process this, and I'm happy to talk more when you're ready."*

The following case studies can be tailored to the unique experiences of MSM, helping them disclose their sexuality.

Case study 3: Non-disclosure and in the closet

Cognitive Restructuring

Goal: Challenge and change unhelpful beliefs or thoughts.
Situation: Peter was struggling with mental health issues as he was bullied at work. This started in his first job and has, even as he left this job, surfaced again and again in any new employment. Peter knew he was different, but he couldn't share it with his mother and siblings. His father was from Mozambique and had long gone. He felt overwhelming guilt and shame about being gay.

- **Negative Thought:** "I'm a failure because I love men. No one will ever love me."
- **Cognitive Restructuring Technique:**
  - **Identify the Thought:** Help him recognize the negative thought.
  - **Examine Evidence:** Ask him to list evidence for and against these thoughts. For example, has he experienced love and support from others? Has he been able to achieve goals despite his status?
  - **Develop Balanced Thoughts:** Replace the negative thought with a balanced statement like, "Loving men doesn't define my worth". "I love and respect myself whoever I love". "I am capable of being loved and being successful".

Case study 4: Disclosure of homosexuality

Self-Compassion Exercises

Goal: Build self-acceptance and reduce self-criticism.

- **Situation:** Bongani is a 27-year old gay graphic designer who lives in Johannesburg where he feels accepted and supported. However, going back home is a challenge due to the conservative views of his family and community. When he returns for his cousin's wedding, Bongani feels the weight of disapproval by his family members and feels intense criticism.

- **Technique:**
  - **Self-Compassion Break:** Teach him to acknowledge his pain, offer himself kindness, and recognize that others also experience similar struggles.

- **Writing Exercises:** Encourage him to write a letter to himself from the perspective of a compassionate friend, focusing on understanding and empathy.

In the second part of this module you will get information about and key messages for HIV medication, including Antiretrovirals (ARVs) and Pre-exposure Prophylaxis. (PrEP).

The following key points will help you to address:

- The purposes and mechanisms of ARVs;
- The benefits of being on ARVs;
- Adherence and side effects;
- Undetectable = Untransmittable (U=U);
- Monitoring and regular check-ups;

- How PrEP works;
- The effectiveness of PrEP;
- Why to consider using PrEP;
- Side effects;
- Monitoring and maintenance

Learning Objectives

Using the INUKA Problem Solving Method (INUKA PST) as your guidance to

- Assess how your client deals with ARV/PrEP adherence
- Recognize adherence challenges
- Define adherence challenges
- Brainstorm for solutions
- Make a SMART action plan

Goals

1. Applying INUKA PST 1) to explore, tackle, and ultimately prevent adherence issues, and 2) to potentially help your clients define disclosure problems, brainstorm for and evaluate possible solutions, listing the steps needed to execute the situation.

You will make use of key messages to promote understanding, adherence, and the overall well-being of your clients on ART or on PrEP.

Below follows a brief overview of ARVs:

Purpose of taking ARVs and its Mechanism:

- **Suppressing the Virus:** ARVs work by reducing the viral load in the body to undetectable levels, meaning that HIV is still present but cannot be detected by standard tests and is not transmittable.
- **Combination Therapy:** Treatment typically involves **Antiretroviral Therapy (ART)**, which uses a combination of drugs from different ARV classes. This helps prevent the virus from developing resistance.
- **Mechanism:** ARVs target different stages of the HIV life cycle to inhibit replication and reduce the viral load in the bloodstream.

Importance of taking Antiretroviral Therapy:

- **Viral Suppression:** Achieve and maintain an undetectable viral load.
- **Restore Immune Function:** Improve the function of the immune system and increase CD4+ cell counts.
- **Reduce HIV Transmission:** People with undetectable viral loads cannot transmit the virus sexually "U=U"

U=U stands for **"Undetectable = Untransmittable"**. It means that when a person living with HIV is on ART and maintains an undetectable viral load (the amount of HIV in the blood is so low that it cannot be detected by standard tests), they cannot sexually transmit the virus to others.

Benefits of ART:

- **Improved Quality of Life:** ART allows patients to live long, healthy lives comparable to those without HIV.
- **Prevention of HIV Progression:** Reduces the risk of HIV-related illnesses and complications.
- **Reduced Risk of Transmission:** ART lowers the risk of passing HIV to partners and, during pregnancy, from mother to child.

Adherence and Side Effects:

- **Importance of Adherence:** Consistent adherence to ART is crucial to prevent drug resistance and maintain viral suppression.
- **Side Effects:** Common side effects can include nausea, fatigue, and headaches. Long-term effects might include changes in cholesterol, liver function, or bone density.

Guidelines:

- National Guidelines recommend starting ART as soon as possible after an HIV diagnosis to maximize health benefits. Thanks to the current ARTs, HIV has become a manageable chronic disease. However, regular follow-ups and monitoring are essential for effective treatment and to manage any potential side effects.

APPLICATION OF INUKA PST

How does the client deal with the medication adherence?

- Assess how your client deals with adherence.
- Ask how your client sees adherence as a challenge and what he believes about his ability to address this.
- When your client is avoidant, explain how it is much more beneficial to take a rational stance to solve the adherence problem efficiently and how he will benefit from understanding the importance of adhering to this medication.
- You can assess how your client deals with problems in general by asking the following questions:
    - Guide: Have you tried to solve adherence challenges in the past?
    - Guide: What did you do?
    - Guide: How do you feel when you have problems?

Bear in mind that stressful problems are largely determined by a person's generalized beliefs, attitudes, and emotional reactions about problems and one's ability to successfully cope with problems.

Here are some brief counseling messages to support individuals in understanding and adhering to ART:

1. Understanding the Importance of ART:

- *"Taking your ART daily helps control your viral load, which keeps you healthy and prevents HIV from progressing."*
- *"When your viral load is undetectable, it protects your immune system and reduces the chance of passing HIV to others (U=U)."*

2. Adherence and Consistency:

- *"Taking your medication at the same time every day helps maintain a consistent level in your body, which is key for its effectiveness."*
- *"Missing doses can lead to drug resistance, making the virus harder to treat. Set reminders or use pill organizers to help you stay on track."*

3. Managing Side Effects:

- *"Some side effects may occur, especially at the start of treatment, but most get better over time. If side effects worry you, speak with your healthcare provider for support."*
- *"Communicating openly about side effects can help your doctor adjust your treatment plan to keep you comfortable and healthy."*

4. Regular Monitoring and Check-Ups:

- *"Regular blood tests are essential to monitor your viral load. This helps ensure that your ART is working effectively."*
- *"Routine check-ups help catch any issues early and keep your treatment on track."*

5. Benefits of Adherence:

- *"Consistent ART use can lead to a normal life expectancy and allows you to live a healthy, active life."*
- *"Adherence not only benefits your health but also supports public health by preventing new infections."*

6. Alcohol and substance use:

- *"There are no significant interactions between alcohol, substance use and ARVs"*
- *"It is essential that you keep on taking your medication, even though you drink or use substance"*

7. Support and Resources:

- *"You're not alone. Reach out to support groups, friends, or healthcare professionals for encouragement and help in managing your treatment."*
- *"If you're struggling with adherence, there are tools like mobile apps, daily alarms, or friends who can help remind you to take your medicine."*

(See also Case study 1)

Now follows a brief overview of PrEP:

PrEP (Pre-Exposure Prophylaxis) is a preventive treatment for individuals at high risk of contracting HIV. It involves taking antiretroviral medication to reduce the risk of infection before potential exposure to the virus.

Key Points About PrEP:

1. **How PrEP Works:**
    - Combined in a single pill called Truvada to prevent HIV from establishing an infection in the body.

    - The medication needs to be taken daily to maintain effective levels in the bloodstream, although some regimens and studies are exploring on-demand use, and even “slow release” injectables.*
2. **Effectiveness:**
    - When taken consistently as prescribed, PrEP can reduce the risk of HIV transmission through sexual contact by almost 100%.
    - PrEP does not protect against other sexually transmitted infections (STIs) or pregnancy, so additional preventive measures, like condoms, are recommended for comprehensive protection.
3. **Who Should Consider PrEP:**
    - PrEP is recommended for individuals who are at higher risk of HIV exposure, including:
        - People with an HIV-positive partner.
        - Individuals who have multiple sexual partners or engage in sexual activity without condoms.
        - People who practice anal sex
        - People who inject drugs and share needles or other injection equipment.
        - Those who have been diagnosed with an STI in the past six months, indicating increased vulnerability.
4. **Monitoring and Maintenance:**
    - Regular follow-up appointments are essential while on PrEP, typically every three months, to monitor HIV status, side effects, and overall health.
    - These appointments include HIV testing, kidney function checks, and discussions about any side effects or challenges with adherence.
5. **Side Effects:**
    - PrEP is generally well-tolerated, but some users may experience mild side effects such as nausea or headaches, which often subside within two weeks. Serious side effects are rare.

Here are some key PrEP messages to inform, motivate, and support clients to better adhere to PrEP.

1. General Awareness:

- *“PrEP is a daily pill that can reduce your risk of getting HIV by almost 100% when taken consistently. It’s a powerful tool for staying HIV-negative.”*

2. Adherence and Consistency:

- *“For PrEP to be most effective, take it every day as prescribed. Skipping doses can reduce its ability to protect you from HIV.”*

- *"Set reminders or use apps to help you remember to take PrEP daily—consistency is key!"*

3. Encouragement and Support:

- *"If you're using PrEP, know that it's a safe and effective way to take control of your sexual health."*
- *"Being on PrEP is a responsible choice for protecting yourself and your partners. Keep up with your routine and stay informed."*

4. Follow-Up and Monitoring:

- *"Regular check-ups are an essential part of being on PrEP. Schedule follow-up appointments every three months to ensure your health is on track."*
- *"Your doctor will monitor your health, conduct HIV tests, and check for any side effects or kidney function to keep you safe while on PrEP."*

5. Prevention Beyond PrEP:

- *"PrEP works best when combined with other prevention measures like using condoms and getting regular STI screenings."*
- *"Remember, PrEP protects against HIV but not other STIs. Practice safe sex to ensure comprehensive protection."*

6. Stopping PrEP:

- *"If you're thinking of stopping PrEP, consult your doctor to understand how to safely transition off and explore other prevention options."*

7. Community and Accessibility:

- *"There are programs that can assist you to access and stay on PrEP.*

Case study 5: HIV status disclosure, and being on PrEP

1)Mindfulness-Based CBT

Goal: Develop mindfulness skills to manage stress and anxiety.
Situation: Lubanzi learns indirectly that his boyfriend of 6 months was unfaithful and recently tested HIV positive. Lubanzi doesn't know how to discuss the topic of HIV status disclosure and decides to get tested himself. He tests negative and starts on daily oral PrEP. Affected by mistrust and fear, he doesn't hide the PrEP bottle at home. Both partners accuse each other of being unfaithful and their quarrels intensify till the point that Lubanzi's boyfriend leaves him.
Now, Lubanzi worries all the time about any unfaithful relationship, about the world at large, and that he himself would wake up one day and test HIV positive.

- **Technique:**
    - **Mindfulness Exercises:** Teach him to focus on the present moment through mindfulness meditation or breathing exercises.
    - **Thought Observation:** Encourage him to observe his thoughts without judgment and recognize when he's ruminating on negative outcomes.
    - **Acceptance Practice:** Help him practice accepting uncertainty and the limitations of what he can control regarding his health.

2)Behavioral Activation

Goal: Increase engagement in positive activities to counter depression.
Situation: Technique:

- **Identify Activities:** Work with him to identify activities he enjoys or used to enjoy (e.g., exercise, attending support groups, or hobbies).
- **Create an Activity Schedule:** Gradually reintroduce these activities into his routine, starting with manageable steps.
- **Reflect on Feelings:** After each activity, discuss how it made him feel and any changes in mood.

*Extra information about PrEP Injectables:

PrEP injectables are an alternative for oral PrEP.

1. **Mechanism and Use:**
    - PrEP injectables use **cabotegravir** that prevents HIV from replicating within the body.
    - Administered as an intramuscular injection every two months, this long-acting formulation helps reduce the need for daily oral medication, making it more convenient for users.
2. **Efficacy:**
    - The injections provide consistent medication levels, ensuring protection.
3. **Target Populations:**
    - PrEP injectables are particularly beneficial for individuals who struggle with daily pill adherence or prefer a less frequent regimen. This includes those at higher risk for HIV, such as men who have sex with men (MSM), transgender individuals, and sex workers.
4. **Administration and Follow-Up:**
    - The first dose is given as an injection after an initial oral lead-in phase, which may help identify any adverse reactions to the medication.
    - Regular follow-up appointments are necessary for ongoing injections and health monitoring.
5. **Safety and Side Effects:**
    - PrEP injectables are generally safe, with mild to moderate side effects such as pain at the injection site, headaches, or fatigue.

    - Long-term studies have shown no significant adverse effects, making injectable PrEP a promising addition to HIV prevention strategies.
6. **Approval and Availability:**
    - **Cabotegravir for PrEP** has been approved in various regions, including the U.S. and parts of Africa. It is being rolled out in select countries and is expected to become more widely available as production and distribution expand.

Benefits of Injectable PrEP:

- **Improved Adherence**: Reduces the burden of daily medication, leading to better adherence and consistent protection.
- **Long-Lasting Protection**: Provides up to two months of HIV prevention with each injection.
- **Reduced Stigma**: For some, using injectables can decrease the stigma associated with taking daily pills, especially in environments where taking PrEP may be misunderstood.

Challenges:

- **Access and Cost**: PrEP injectables can be more expensive and may not be accessible everywhere due to logistical and cost-related barriers.
- **Healthcare Visits**: Requires regular clinic visits for injections, which may be a limitation for some users.

PrEP injectables are not yet widely available. Broader access and awareness is essential to ensure that more people benefit from this innovative prevention method.

How to manage INUKA MENTAL HEALTH & HIV MEDICATION ADHERENCE (ART/PrEP) together:

I MENTAL HEALTH & MEDICATION ADHERENCE

Address mental health and medication adherence directly by discussing results of SRQ, (PHQ-9?), and viral loads (elevated) or dry blood spots for PrEP presence.

Please, note that by discussing the results, sensitivity, respect, and privacy are required. Here are some tips how you can approach it.

Tips for asking:

1. Start with open-ended and non-intrusive questions:

o "Can you share what coping mechanisms you usually use to manage stress?

o "What strategies do you use to manage ART / PrEP adherence?"

o "Can you share what your experience with healthcare has been like?"

o "What kinds of support or services have been most helpful for you in managing your health?"

2. Focus on client's comfort:

o "If you are comfortable sharing, how do you usually access HIV treatment (ART or PrEP), and care?"

o "What has worked well for you in finding the right healthcare providers, services and support?"

Express understanding and support:

o "Managing health can be complex—how do you find care that meets your needs?"

o "What kind of resources or care do you feel are most important for you?"

Things to avoid:

· Avoid making assumptions or judgments.

· Ensure your tone is non-invasive and shows genuine interest in clients' well-being.

This approach fosters trust, allowing your client(s) to share his (their) experience if he (they) feel comfortable.

II SOCIAL SUPPORT

Ask about social support in an empathetic way involves creating a safe, non-judgmental environment and expressing genuine concern.

Here are some examples of how to approach this:

1**. Show care and openness:**

o "I know having support can make a big difference. Are there people or groups you turn to for help or encouragement?"

o "It's okay if this is personal, but I'm here to listen if you want to share about the kind of support you have or need."

2. Acknowledge client's experience:

o "Dealing with these challenges can be overwhelming. Do you have someone you feel comfortable talking to or leaning on?"

o "Having someone to talk to about these things can help a lot. Is there anyone you feel you can rely on?"

3. Offer assistance in a gentle way:

o "Sometimes, reaching out for support isn't easy. Would you like help finding resources or groups that could support you?"

o "It's completely normal to need a little extra help. Are there specific ways others could support you better?"

4. Focus on client's needs and comfort:

o "Everyone needs support sometimes, and it looks different for each person. What kind of support do you feel would be most helpful for you?"

o "You deserve care and understanding. What does support look like for you right now?"

5. Be respectful of boundaries:

o Avoid pushing if they're not ready to share. You might say:

"If you're not ready to talk about this, that's okay. Just know I'm here whenever you need someone to listen."

These approaches balance curiosity with empathy and respect, fostering trust and encouraging open communication.

III STIGMA

In line with the discussion about "Social Support", you can address possible "Stigma".

Ask how the client can be more supported when he is worried about stigma around HIV or his sexuality.

Note that supporting someone who is worried about stigma around HIV and homosexuality requires empathy, reassurance, and practical assistance.

Here are some questions to offer meaningful help:

1. Try to provide emotional support:

Validate client's feelings and acknowledge their concerns without judgment.

o "How do you feel about stigma around HIV / being HIV-positive / being gay?"

As coach you may say: "I understand that you are worried. It can be tough, but you're not alone in this."

Reassure the client and remind him that their identity and health status do not diminish their worth.

As coach you may say: "Being HIV-positive or identifying as gay doesn't define your value as a person. You deserve respect and care."

2. Reassure a safe space:

Offer a judgment-free environment where they feel comfortable sharing their thoughts and fears.

o "How can I be more supportive?"

As coach you may say: "You can always talk to me about how you're feeling. I'm here to support you, no matter what."

Avoid assumptions or intrusive questions about their health or sexuality.

3. Encourage connection to support systems:

o "Have you thought of joining a group where others understand what you're going through?"

As coach you may say: "Have you thought of connecting with LGBTQ+ or HIV-positive peer support groups where you can share experiences and build community?"

Or

Recommend culturally competent therapists or counselors who are experienced in LGBTQ+ and HIV-related issues (Pop Inn)

4. Address stigma directly:

"Can the client share instances that stigma stemmed from ignorance or fear?".

As coach you may say: "Stigma is a reflection of society's lack of understanding, not a reflection of you."

o Show acceptance and support in public conversations to help normalize discussions around HIV and homosexuality.

5. Help with practical resources:

Access to Care: Support the client in finding LGBTQ+ and HIV-specialized healthcare providers if needed.

o Example: "Can I help you look for clinics or doctors who provide nonjudgmental and inclusive care."

o Legal protections: Make the client aware of laws and rights that protect them against discrimination in their area.

6. Reframe client's focus:

Emphasize personal strengths and resilience.

o "Your courage in facing this shows your strength. You're more than the stigma society may place on you."

o Highlight how treatment, such as ART and PrEP, and self-acceptance can empower them.

7. Be Patient and Persistent:

Support client's pace. He may need time to process his feelings and fears.

Continue to check in with your client and remind him that are not alone.

Providing consistent, affirming support can help them navigate the challenges of stigma while fostering confidence and resilience.

IV MAKING ADHERENCE SUSTAINABLE OVER TIME

Discussing improvement of medication adherence involves small, practical steps that focus on reducing risks while respecting client's autonomy.

Here are some careful steps to consider:

1. Build trust and understanding:

Listen without judgment: Start by understanding why adherence is challenging.

o "Can you share how you feel about taking your medication? I'm here to help, no matter what."

Acknowledge client's efforts:

o "It's great that you're thinking about your health. Every small step matters."

2. Set realistic goals together:

Start small: Encourage small, manageable changes rather than perfection.

o “Would taking your medication at the same time each day feel more doable? We can find a time that works for you.”

Track progress together: e.g. celebrate milestones, such as taking medication consistently for a week.

3. Simplify client’s routine:

Tailor to client’s lifestyle:

o “What would work with the client’s schedule to find the least disruptive time for taking medication?”

o “Would it help to set a reminder on your phone so it’s easier to remember?”

4. Address barriers incrementally:

Side Effects: If they mention discomfort, help them discuss solutions with a healthcare provider.

o “Have you noticed anything bothering you when you take the meds? Maybe the doctor can suggest an adjustment.”

Access Issues: Assist with finding affordable or nearby pharmacies or transportation options.

o “What services can make it easier for you to get refills?”

5. Reframe adherence positively:

Focus on benefits: Frame adherence in terms of achieving personal goals (e.g., better health, staying active).

o “Do you think taking your meds regularly can help you feel stronger and more in control?”

Address resistance gently: Normalize occasional struggles.

o “It’s okay to miss a dose sometimes. Let’s find ways to make it easier tomorrow.”

6. Create a Support System:

o “Would you like me or someone else to check in with you now and then to see how it’s going?”

o “Do you think that a case manager, counselor, or adherence specialist should check-in for regular monitoring your adherence”

7. Incorporate Harm Reduction:

Partial adherence: If full adherence feels overwhelming, encourage taking as much as possible rather than stopping completely.

o “Even if you miss a dose, taking the next one as planned is still helpful.”

Combine with other support: Help the client manage stress, sleep, and nutrition, which can indirectly improve adherence.

8. Monitor progress and adjust:

Check in periodically to identify new barriers and adjust strategies.

o "How's it going with your meds? Are there ways to make it even easier for you?"

Case study 6: Cultural and religious conflicts impacting medication adherence

Cognitive Restructuring

Goal: Address intersection of health, faith, and mental well-being.
Situation: Lethabo is a 30-year old gay man who lives with HIV and decided to stop taking his ART medication after the religious leader of his church convinced him that prayer and trust in God's power can cure him completely. Lethabo, who deeply values his religious beliefs, is torn between medical advice from his healthcare provider and the spiritual guidance from his religious community. He suffers from feelings of constant unworthiness, but remains reluctant to return to treatment due to the fear of judgment from his faith community and a strong internalized belief that seeking medical help indicates a lack of faith.
Technique: Use Motivational Interviewing (MI) to address and discuss the following:
1.Acknowledge and validate Lethabo's beliefs.
Begin by affirming the importance of his spirituality. Express understanding of how deeply held beliefs can shape health decisions.

- *"It's clear that your faith is an important part of your life, and I respect your commitment to it."*

Normalize internal conflict.
Validate the difficulty of reconciling faith with medical advice.

- *"It's understandable to feel torn between the guidance of your faith community and the recommendations from your doctor."*

2.Provide psychoeducation.
Reframe medical treatment in a faith context.
Explain that ART can be seen as a tool provided by God to promote healing and health.

o *"Many people see medicine as a way God works through science to help us heal. Taking care of your health is a way of honoring the body God has given you."*

Clarify the role of ART: Share simple, non-technical information about how ART works to suppress the virus and prevent illness.

o *"ART helps your body stay strong so you can live fully and continue to practice your faith."*

3. Discuss feelings of unworthiness.
Explore the root of these feelings:
Use open-ended questions to understand why Lethabo feels unworthy.

o *"Can you share more about what makes you feel this way? What does your faith say about worthiness?"*

Reaffirm his value:
Highlight the unconditional value of his life, drawing on affirming religious teachings if appropriate.

- *"Your faith teaches that every life is precious. Seeking help for your health shows strength and courage, not weakness."*

4. Navigate together the fear of judgment.
Identify safe allies:
Encourage Lethabo to identify people in his faith community who are supportive or might share similar views on combining faith with medicine.

- *"Is there someone in your church or another faith leader you trust who might help you find balance in this?"*

Explore anonymity:
If stigma is a concern, suggest ways to access medication discreetly (e.g., home delivery services).

5. Elicit Lethabo's own reasons for his health.
Focus on his goals, such as continuing to participate actively in his faith or taking care of loved ones.

- *"How does being healthy help you fulfill your role in your community and family?"*

Resolve ambivalence:
Help Lethabo explore the consequences of stopping ART versus resuming treatment.

- *"What do you think might happen to your health if you continue without medication? How would that affect your ability to pursue your faith and goals?"*

6. Encourage gradual steps.
Start small: Suggest a trial period of returning to ART while continuing his spiritual practices.

- *"Would you be open to taking your medication again and seeing how it affects your health and your faith journey?"*

Integrate prayer and treatment:
Encourage him to view ART as complementary to prayer, not contradictory.

7. Offer supportive resources.
Faith-based support groups: Connect Lethabo with organizations or groups that integrate faith and HIV care, such as faith-led HIV initiatives or counseling services.
Counseling: Recommend a counselor experienced in addressing health-related stigma and religious conflicts.

8. Monitor progress and provide follow-up.
Check in regularly to address ongoing fears, reinforce positive changes, and adjust the approach as needed.

Sample Transcript – Edited from Pilot in 2023 to Include HIV MA module

Sender name	Message
Coach	A warm welcome to Inuka! I am coach X and I will be guiding you as your Inuka coach in the upcoming session(s)
Coachee	Hi coach X.
Coach	I am sitting in a comfortable place and I am ready to start the session, how about you?
Coachee	I'm comfortable and looking forward to the session
Coach	Great, please allow me start by introducing you to the structure we will be following for your(/ these) sessions. The first session is 90 minutes and the following sessions are 60 minutes. On average we will have 4 sessions in total.
Coachee	okay
Coach	In these chat / (In todays) session(s) we will look into(at) your HIV status, how you are/you have been feeling and taking your medication as well as the life challenges you are/ have been experiencing. We will try to understand them a little better and break them down. The goal is to focus on your health and have a clear plan to tackle one life challenge at a time /(in every session).
Coachee	ok, sounds good
Coach	Do you have any questions for me up until this point?
Coachee	No, pretty clear so far
Coach	Ok, thank you. Our conversations will all be through this chat platform, they are anonymous and confidential, So feel free to share.
Coachee	ok
Coach	We will also review todays action points in our next session
Coachee	ok
Coach	Great, thank you. As we proceed with the session, I am curious to hear what your expectations regarding the coaching / this session are?
Coachee	Well, I'm hoping this will help me feel better. Lately, I've been feeling off balance
Coach	thank you for sharing your expectations with me. Lets explore abit more on what has been contributing to how you are feeling.
Coachee	Ok.
Coach	Perhaps we can start with your health and look at the results of your wellbeing scan. This is the set of questions you answered before the session. Your score is 5. which indicates that challenges are weighing on you. Life is not easy for you right now. While you are coping, you run the risk of getting burned out or more distressed. How do you feel about this score and what has contributed to it?

Coachee	yes, It's alot harder to cope now days. Well, I am HIV positive, I was diagnosed about 7years ago, it was a tough diagnosis, but, I found a way to deal and keep living.
Coachee	however, lately I have been feeling lost and stuck
Coachee	I am tired of taking my medication, so many pills in one day, sometimes I feel better then I feel worse... the fluctuations are too much for me at the moment
Coachee	I feel like my life is not moving like I'm stuck, I don't know if I'm making any sense
Coachee	I want a better job, a bigger house, I would like to find a partner but with my status its very scary
Coachee	I don't go out at all not since my diagnosis and I miss just feeling free and happy, I miss my friends and how full of life I used to be.
Coach	Thank you for sharing this with me, you mention that the fluctuations are abit too much for you at the moment. Are you still taking your ARVs?
Coachee	Yes, I am. Though I really hate them and sometimes I forget.
Coach	Taking your ART daily helps control your viral load, which keeps you healthy and prevents the HIV virus from progressing. When your viral load is undetectable, it protects your immune system and reduces the chance of passing HIV to others. Every medication has side effects some good others not so much. Have you spoken to your doctor about your current viral load and the side-effects you are having from the medication?
Coachee	Honestly, I haven't spoken to anyone about it. Its been 7 years and I still feel shame everytime I have to fill my prescriptions and even though I don't feel my best I keep taking my medication because I fear the painful death associated with the virus. I have my viral load tests done get my updates and leave as fast as I can.
Coach	I see, thank you for opening up to me on this. How often would you say you have blood tests for your viral load?
Coachee	I've probably done 5 or 6 blood tests since my diagnosis
Coach	Ok, the reason I ask is because the viral load dictates your prescription and this my be the reason you have fluctuating side effects.
Coach	If I am taking a bit longer to reply, it's because I am making notes. Don't worry, I can still see your messages.
Coachee	Ah I see!! So it's possible I may not be on the right dose and that's why I'm fluctuating a lot?
Coach	Yes, it is possible. I would like to commend you for taking your meds even when you didn't feel that great on them. Would you be open to speaking to one of our HIV specialists at one of our clinics? The program that connected us is specifically focused on mental wellbeing and HIV medication adherence our aim is to help get you on a healthy wellness schedule/ structure. Our specialists are kind and confidential. Would you be open to it?
Coachee	Honestly, I don't know, I will think about it though.

Coach	That's great, thank you for the consideration. I will place the clinic contact information below so it easy for you to access whenever you are ready.
Coachee	Thank you.
Coach	How are you feeling so far?
Coachee	Honestly, not sure, I think I need to go get a blood test and get on the right prescription but I feel scared over what the tests will show. Though I think I just need some time to think about this.
Coach	You've done a great job so far. I appreciate your honest response. In- light of it. Would you like us to stop here or maybe talk about one of the other things contributing to the wellbeing score you got?
Coachee	I would like to continue
	Ok, great.<br><br>I was taking some notes for you and aimed to capture everything you have shared in a list of "Things going on in my life". Let me share this with you "things going on in my life'<br>• I've been feeling off balance<br>• I am HIV positive, diagnosed about 7years ago.<br>• I have been feeling lost and stuck<br>• Tired of taking my medication, the fluctuations are too much for me at the moment.<br>• Feel like my life is not moving like I'm stuck.<br>• I want a better job, a bigger house,<br>• I would like to find a partner but with my status its very scary<br>• I don't go out at all not since my diagnosis<br>• I miss just feeling free and happy<br>• I miss my friends and how full of life I used to be
Coach	Looking at this list, is there anything missing or something that should be changed?
Coachee	No, you have captured everything so far
Coach	How are you doing with regards to sleep and family?
Coachee	I'm sleeping ok, I don't have any family it's just me
Coach	Our next the next step is to choose 1 item on the list that you want to work on. what would you like to focus on next?
Coachee	Maybe finding a partner, I'm so tired of being alone. You had mentioned there is a reduced chance of me passing the HIV virus if on the proper medication and viral load?
Coach	Yes, I had. Though closely working with your doctor, taking your medication as prescribed and looking after yourself it is a possibility.
Coachee	That's amazing how did I not know this. I thought I was only limited to HIV+ partners and with all the stigma I didn't want to risk it.
Coach	Sounds like this has been something you have thought about a lot. Tell me what would change or how would your life be different should you find yourself with a partner?

Coachee	I hope for a good partner who I can be happy with, share experiences with and someone who would accept me as I am.
Coach	Would it be ok for us to set this as your goal for todays session?
Coachee	Yes
Coach	"The Change I want to see"<br>I hope for a good partner who I can be happy with, share experiences with and someone who would accept me as I am.
Coach	Lets dive a little deeper into your goal.<br><br>Have you dated anyone since your diagnosis 7 years ago?
Coachee	I wouldn't say I have. I ex who was my then partner infected me with HIV when I found out I felt so much pain and hurt but also trapped. How could I date someone else and risk infecting them, do to them what was done to me. So I stayed with my ex for another 3 years post diagnosis.
Coach	Thank you for sharing this with me. If I may ask what were those 3yrs post diagnosis and in the relationship like for you?
Coachee	Honestly it was hell on earth. I feel like I lost myself and my partner never stopped cheating with everyone he could find. I honestly think he was on a mission to infect everyone he could. He was abusive and absent, did whatever he wanted whenever he wanted. So one day I had enough decided it was easier to live alone with my diagnosis than to keep being in a relationship with him.
Coach	I can only imagine what that period was like for you. Thank you for trusting me with your story. How are you feeling so far?
Coachee	You are welcome. I am ok.
Coach	How about after the break up. Did you date anyone from then to now?
Coachee	No, I was too afraid of infecting someone or dating another person with HIV who might just be like my ex.
Coach	I see. Well, lets see how we can help you get to your goal today. We call it the brainstorming session.<br><br>What do you think would be the best ways to meet people and find a potential partner?
Coachee	I honestly don't know.
Coach	That's ok. Lets role play the question. If a friend approached you and told you they have been HIV+ and looking to start dating again, where would you advise them to start?
Coachee	Hhmmm, I would suggest they be bold and try some new things that feel safe for them
Coach	Ok, like what ?
Coachee	Maybe a dating app but only do lunch or coffee dates during the day for safety, maybe go for a party or two or try some group outdoor activities like hikes or walks to meet new people.

Coach	Those are amazing suggestions. Would you be willing to try them yourself?
Coachee	Actually, maybe yes. They sound like something I would do too.
Coach	Great, anything else coming to mind as to how you can achieve this goal?
Coachee	No
Coachee	Well actually, would reaching out to old flings be ok, like talking to people who used to want to date you or something?
Coach	That depends on what you are comfortable with and what you would be willing to try. Should I add it to the list for you?
Coachee	Yes please.
Coach	"Things I could Try"<br>• A dating app but only do lunch or coffee dates during the day for safety.<br>• Maybe go for a party or two<br>• Try some group outdoor activities like hikes or walks to meet new people.<br>• Reaching out to old flings
Coach	Is there anything else you would like me to add to the list?
Coachee	No. that's everything I can think of at the moment.
Coach	Ok great. What do you feel like you can try in the next week from the list above?
Coachee	I thin option 1 – the dating app
Coach	Ok, you've been doing great so far, and we are coming to the end of our session. But before we close, why option 1 ?
Coachee	Because I can start and control the conversations without meeting anyone, it feels safer for me since if I don't like a conversation, I could always log out without consequences of feeling guilty or shame.
Coach	You have really though this out.
Coach	What steps do you plan on taking to get onto the app?
Coachee	Well, I think I will have 2, a paid one and a free one that are MSM friendly, Maybe look for a cute picture of myself, only one for now. Then see what happens.
Coach	Ok, sounds like a plan. When would you like to join the apps?
Coachee	Maybe this evening or tomorrow.
Coach	That's great. Do you plan to meet anyone in the next 2 weeks if the opportunity presents its self? Do you need any help setting up?
Coachee	No, I feel like 2 weeks is a very short time for me at the moment, I also don't think I need any help.
Coach	Ok, our time is running out would you be comfortable with me adding 10mins to our session so we can finalize?
Coachee	Yes, That's fine
	10mins Added to session
Coach	I've also aimed to capture the steps you intend to take to help you achieve your goal.<br>"Steps I Will Take"<br>• I will have 2 apps, a paid one and a free one that are MSM friendly<br>• Maybe look for a cute picture of myself, only one for now

	• Join the apps this evening or tomorrow
Coach	All the notes taken for you in todays session have helped ups build an action plan for you. You can access this at anytime on your home page and use it to trach your progress or even review some note as you go along.<br><br>Action Plan sent
Coachee	Thank you very much.
Coach	Would you be open to scheduling another session in the next 14 days?
Coachee	Yes I would.
Coach	That's fantastic, what day and time work best for you?
Coachee	Same time Friday Next week.
Coach	Perfect, I will schedule the session for us. In our next session we will review the action plan from todays session and perhaps talk abit on status disclosure and any other item on the list shared.
Coachee	That sounds like a plan
Coach	Great. For now I hope you enjoyed your session, do you have any feedback for me?
Coachee	I did thank you, I feel lighter. I have a lot to think about now but I'm hopeful. Thank you
Coach	Hopeful is good. Thank you for the feedback. Until next time, bye for now.
Coachee	Bye
